# Editorial: *In vivo* functional neuroimaging for pharmacology

**DOI:** 10.3389/fnins.2023.1279587

**Published:** 2023-09-21

**Authors:** Benjamin Vidal, Luc Zimmer

**Affiliations:** ^1^Lyon Neuroscience Research Center, Lyon, France; ^2^CERMEP-Imaging Platform, Bron, France

**Keywords:** functional ultrasound (fUS), PET, fMRI, LFP, CNS, pharmacology

Currently available pharmacological treatments for many neuropsychiatric diseases still have limitations in terms of efficacy and safety. This necessitates the exploration of new approaches in drug development for central nervous system (CNS) disorders. Developing efficient CNS drugs has been recognized as highly challenging due to several reasons, including the difficulty in predicting brain penetration of drug candidates, their molecular binding to targets, and the resulting functional activation. In this context, functional neuroimaging emerges as a promising method to address these challenges by monitoring brain activity and its changes following drug administration. Technological advancements over the years have enabled the probing of brain activity at multiple spatiotemporal scales, providing valuable insights into drug effects in both living animals and humans. Functional neuroimaging offers a way to identify the most promising compounds and improve our understanding of drug mechanisms of action in pathological contexts. These contributions are illustrated by the articles selected for this Research Topic, in which pharmacological questions are addressed using different *in vivo* neuroimaging modalities, i.e., positron emission tomography (PET), functional magnetic resonance imaging (fMRI), functional ultrasound imaging (fUS) and intracerebral electrophysiology.

PET is a highly translatable, non-invasive imaging method widely used in pharmacological research for target engagement, biodistribution, and biomarker measurements. Despite its relatively high cost, PET's contribution to neuropharmacology through *in vivo* imaging at a molecular level is well-established. However, in preclinical small-animal imaging, fully quantitative PET imaging to access ligand concentration or receptor occupancy can be challenging due to limited access to radiopharmaceuticals and methodological constraints related to radiopharmacology and radioactive metabolites. Radiolabeled fluorodeoxyglucose (^18^F-FDG) offers a solution to these issues and provides insights into the metabolic profile and potential mode of action of pharmacological agents. Two papers in this Research Topic investigated the use of ^18^F-FDG as a “surrogate treatment response” marker for buprenorphine and ketamine. Soyer et al. showed that regions with the highest post-buprenorphine decrease in normalized ^18^F-FDG signal coincided with the regions with the highest buprenorphine binding. This study opens possibilities for detecting longitudinal and intrasubject variability in responses to opioids by measuring changes in regional brain glucose metabolism. The second paper by Chaib et al. explored longitudinal changes in ^18^F-FDG uptake following a single injection of a subanesthetic dose of ketamine and suggested a potential “biosignature” of ketamine-like antidepressants.

In a complementary manner, fMRI has been utilized for many years as a tool for deciphering the impact of drugs on brain activity. Using a drug directly as a stimulus to induce hemodynamic changes, known as “pharmaco-MRI,” allows for easy exploration of changes in the whole brain with a temporal resolution in the order of seconds. Recent advancements in fMRI have enabled imaging of awake, restrained animals, eliminating the confounding variable of anesthesia. Brems et al. evaluated the effects of a positive allosteric modulator of α7 nicotinic receptors, at three doses in awake rats. The authors showed a non-linear effect of the drug with maximal size of BOLD changes in numerous regions at the intermediate dose and a global decrease of functional connectivity, shedding new light on the drug's action mechanism.

A recent neuroimaging technique, functional ultrasound imaging (fUS), relies on hemodynamics and allows the measurement of cerebral blood volume changes at high spatiotemporal resolution. Several proof-of-concept studies demonstrated its value for neuropharmacological applications. Ionescu et al. utilized fUS to study changes in functional connectivity patterns occurring in different frequency bands after the administration of ketamine. The authors demonstrated that ketamine induced transient increases in connectivity in all frequency ranges, except for the fastest frequencies, which displayed decreased functional connectivity in corticostriatal areas. This specific effect persisted for 9 days after the beginning of the treatment.

Intracerebral electrophysiological recordings, despite being invasive and limited in their field of view, provide direct insights into the electrical activity of neurons, which are crucial for understanding psychoactive compounds. Nasretdinov et al. conducted large-scale multi-structure microelectrode local field potential (LFP) recordings in freely-moving rats treated with LSD, ketamine, or the non-hallucinogenic amphetamine. The authors primarily focused on the aperiodic part of LFP, associated with asynchronous spiking rather than synchronized currents and linked to the fMRI signal. Their findings revealed distinct brain activation patterns induced by the compounds: ketamine resulted in a substantial increase in spectral power, while LSD predominantly triggered an increase in the excitation/inhibition balance.

Finally, this Research Topic illustrates the dynamism of research in psychopharmacology which is evolving through the use of revisited psychotropic molecules and the integration of new exploration dimensions and experimental paradigms brought about by neuroimaging technologies. Neuroimaging, including PET, fMRI, fUS, and intracerebral electrophysiological recordings, provides valuable tools for advancing pharmacology in the CNS and improving the development of drugs for neuropsychiatric diseases.

## Author contributions

BV: Writing—original draft. LZ: Writing—review and editing.

